# Does pain self-efficacy predict, moderate or mediate outcomes in people with chronic headache; an exploratory analysis of the CHESS trial

**DOI:** 10.1186/s10194-024-01768-5

**Published:** 2024-05-15

**Authors:** Siew Wan Hee, Shilpa Patel, Harbinder Sandhu, Manjit S. Matharu, Martin Underwood, on behalf of the CHESS Team, Felix Achana, Dawn Carnes, Sandra Eldridge, David R. Ellard, Frances E. Griffiths, Kirstie Haywood, Siew Wan Hee, Helen Higgins, Manjit S. Matharu Dipesh Mistry, Hema Mistry, Sian Newton, Vivien P. Nichols, Chloe Norman, Emma Padfield, Shilpa Patel, Stavros Petrou, Tamar Pincus, Rachel Potter, Harbinder Sandhu, Kimberley Stewart, Stephanie J. C. Taylor, Martin Underwood

**Affiliations:** 1 University Hospitals Coventry & Warwickshire NHS Trust, Clifford Bridge Road, CV2 2DX Coventry, UK; 2 Warwick Medical School, University of Warwick, CV4 7AL Coventry, UK; 3 Queen Square Institute of Neurology, University College London, Queen Square, WC1N 3BG London, UK

**Keywords:** Behavioural therapy, Chronic migraine, Chronic tension type headache, Education, Episodic headache, Mediator, Medication overuse, Moderator, Predictor, Self-management

## Abstract

**Background:**

Chronic headache disorders are disabling. The CHESS trial studied the effects of a short non-pharmacological intervention of education with self-management support for people affected by migraine and/or tension type headache for at least 15 days per month for at least three months. There were no statistically significant effects on the Headache Impact Test-6 (HIT-6) at 12-months. However, we observed improvement in pain self-efficacy questionnaire (PSEQ) and short-term HIT-6. We explored the impact of the CHESS intervention on PSEQ, and subsequently, on the HIT-6 and chronic headache quality of life questionnaire (CH-QLQ) at four, eighth and 12 months.

**Methods:**

We included all 736 participants from the CHESS trial. We used simple linear regression models to explore the change of HIT-6 and CH-QLQ with treatment and PSEQ at baseline (predictor analysis), and the interaction between treatment and baseline PSEQ (moderator analysis). We considered the change of PSEQ from baseline to four months as a mediator in the mediation analysis.

**Results:**

Baseline PSEQ neither predicted nor moderated outcomes. The prediction effect on change of HIT-6 from baseline to 12 months was 0.01 (95% CI, -0.03 to 0.04) and the interaction (moderation) effect was −0.07 (95% CI, -0.15 to 0.002). However, the change of PSEQ from baseline to 4-month mediated the HIT-6 (baseline to 8-, and 12-month) and all components of CH-QLQ (baseline to 8-, and 12-month). The CHESS intervention improved the mediated variable, PSEQ, by 2.34 (95% CI, 0.484 to 4.187) units and this corresponds to an increase of 0.21 (95% CI, 0.03 to 0.45) units in HIT-6 at 12-months. The largest mediated effect was observed on the CH-QLQ Emotional Function, an increase of 1.12 (95% CI, 0.22 to 2.20).

**Conclusions:**

PSEQ was not an effective predictor of outcome. However, change of short-term PSEQ mediated all outcomes, albeit minimally. Future behavioural therapy for chronic headache may need to consider how to achieve larger, and more sustained increases level of self-efficacy than that achieved within the CHESS trial.

**Trial registration:**

ISRCTN79708100.

**Supplementary Information:**

The online version contains supplementary material available at 10.1186/s10194-024-01768-5.

## Background

Chronic headache disorders can be profoundly disabling. In comparison to the substantial amount of research on the biological mechanisms of chronic headache disorders, and success in finding drug treatments of proven benefit for chronic migraine [1], there has been rather less focus on, and less success with, treatments addressing the social and psychological components of the disability caused by chronic headache disorders [2]. The international classification of headache disorders does not recognise the disorder of ‘chronic headache’ as a distinct disorder. Nevertheless, an epidemiological definition of chronic headaches, headaches on 15 or more days per month for at least three months can be used [3–5]. This population predominantly comprises individuals with chronic tension-type headache, chronic migraine, and those experiencing chronic tension-type headache alongside episodic migraine, all of which may occur with or without medication overuse.

A 2017 systematic review identified just four qualitative studies (*n* = 73) of people living with chronic headache [6]. A 2023 systematic review of qualitative studies identified just 10 papers on the experience of living with migraine, two papers (*n* = 36) specifically addressed living with chronic migraine [7–9]. Common themes identified in these studies include; ‘the effect on personal relationships (work/home)’, ‘the difficulty of living with an invisible condition’, and ‘the impact on daily life’ [7–9]. 

We have very little literature on the experience of living with chronic headache disorders to inform suitable targets for non-drug interventions for people living with chronic headache disorders. Nevertheless, there is likely to be considerable overlap between the psychological and social drivers of chronic headache disability and other chronic pain syndromes. For example, a 2019 systematic review (14 studies) found a positive association between chronic headache disorders and persistent low back pain [10]. A 2017 systematic review of prognostic factors for chronic headache (27 studies) found similar prognostic factors for poor outcome in chronic headache to other chronic pain syndromes; including depression, anxiety, poor sleep, stress and poor self-efficacy [11]. Self-efficacy is a person’s conviction in their ability to manage events, situations and reach goals especially when under stress [12]. It is increasingly recognised that the management of primary headache disorder such as migraine needs to be embedded in a biopsychosocial context recognising the overlap with psychological distress and other primary pain disorders [13]. The same authors advocate the promotion of self-efficacy and an internal locus of control as first-line non-pharmacological treatments for migraine [13]. 

In 2022, following a substantial amount of preparatory work we published a randomised controlled trial (*N* = 727) of an education and self-management support intervention for people with chronic headaches (chronic migraine + chronic tension type headache and episodic migraine) [14, 15]; the CHESS trial. The primary outcome was the Headache Impact Test−6 (HIT-6) at 12 months [16, 17]. The observed between group difference at 12 months was −0.3, (95% confidence interval, CI, −1.23 to 0.67) effectively excluding any possibility that the CHESS intervention was effective. The results were not materially different when we analysed the chronic migraine and chronic tension type and episodic migraine groups separately. Our process evaluation did not give any insights as to why our intervention was ineffective [18]. As part of our preparatory work we did a systematic reviewed of multi-item patient reported outcomes for headache disorders [19]. Only the HIT-6 had acceptable evidence for use in a mixed headache population. Consequentially, in our feasibility study we adapted, and validated, the Migraine-Specific Quality of Life Questionnaire (MSQ v2.1) for use in a mixed headache population [20, 21]. We found that the Chronic Headache Quality of Life Questionnaire (CHQLQ), which is reported as three domains, to be structurally valid, temporally stable, internally consistent, and responsive to change with greater relevance to the patient experience when compared to the HIT-6 [20]. Using this in the main trial we also did not find any between group differences.

A specific target of our intervention was improving self-efficacy, with intervention components aimed at informing, empowering and building confidence to implement the self-management strategies [22]. We measured this using the pain self-efficacy questionnaire (PSEQ) [23]. We found positive between group effects on the PSEQ at four months, 2.3 (95% CI, 0.51 to 4.0), favouring education and self-management support intervention, and 12 months 2.1 (95% CI, 0.17 to 3.96), but not at eight months, 1.5 (95% CI, -0.31 to 3.34). If changing self-efficacy has no effect on headache outcomes for people living with chronic headaches, then other targets will be needed for future studies. If on the other hand our observed impact on self-efficacy does impact on longer term headache outcomes, then it may be worthwhile targeting self-efficacy in future studies of non-pharmacological interventions for people with chronic headache disorders. We report here a secondary analysis of the CHESS dataset to explore if PSEQ predicts, moderates and/or mediates headache outcomes at four, eighth and 12 months.

## Methods

The CHESS trial, and its results have been described in detail elsewhere [14, 15, 17, 22]. Briefly, potential participants ≥ 18 years and had consultation for headaches or who had been prescribed with migraine specific drug (triptans/pizotifen) in the previous two years were identified from general practices or self-referred. A research nurse conducted a one-to-one headache classification telephone interview, using a previously validated approach, with those who returned an expression of interest to participate [24]. The headache classification interview allowed us to include participants who met the epidemiological definition of chronic headaches (≥ 15 headache days per month for the past three months). People with ineligible headache types (e.g. cluster headaches), not fluent in English, no access to a telephone, who could not attend or participate in the group interventions were excluded. We recruited 736 people with chronic headaches between 2017 and 2019 to a parallel randomised controlled trial of group supported self-management (henceforth the CHESS intervention) from 166 general practices in England. Nine participants were excluded from our primary analysis because they had chronic tension type headache only. For these analyses we have included all 736 randomised participants to maximise statistical power.

The development and content of the CHESS intervention is detailed elsewhere [22]. Briefly, there were two whole-day group sessions. Day one addressed ‘living, understanding and dealing with chronic headaches’ and day two addressed ‘learning how to adapt and take control of life with chronic headaches’. These were followed by a one-to-one session with a nurse for individual advice, and then continuing telephone support if needed. These sessions included advice on headache classification and use of medications.

The patient-reported outcomes: HIT-6 score ranges from 36 to 78 with higher scores indicating greater severity; PSEQ ranges from 0 to 60 with higher scores suggesting stronger self-efficacy beliefs; and each of the three domains (Role Restrictive, Role Preventive and Emotional Function) of the Chronic Headache Quality of Life Questionnaire (CH-QLQ) ranges from 0 to 100 with higher scores indicating better quality of life [20]. 

We considered four dependent outcomes in each predictor and moderator analyses: change of HIT-6 and change of each CH-QLQ domains from baseline to 4-, 8- and 12-month. The mediator analyses considered the same dependent outcomes but only the changes from baseline to 8- and 12-month. Changes were computed such that positive value indicates improvement. In the predictor analysis, the PSEQ at baseline was the explanatory predictor alongside treatment effect. PSEQ at baseline and its interaction with treatment were considered in the moderator analysis. Simple generalised linear models were used in the predictor and moderator analyses.

**Fig. 1:**
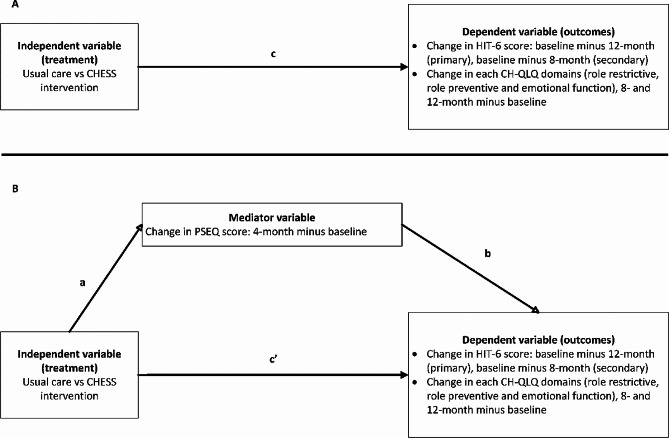
Path diagram of (**A**) total effect between predictor and outcome (simple regression model), and (**B**) mediation model. CHESS intervention was education and self-management support with a one-to-one headache classification interview and advice on drug treatment. The usual care was feedback from headache classification interview with headache management leaflet and a relaxation compact disc

In the mediator analysis, the mediator was the change of PSEQ from baseline to 4-month, i.e. 4-month value minus baseline value. Three separate linear regression analyses were fitted for the mediation analysis (Fig. 1):

1 $$ \varvec{Y}={\varvec{i}}_{1}+\varvec{c}\varvec{X}+{\varvec{e}}_{1},$$

2 $$ Y={i}_{2}+{c}^{{\prime }}X+bM+{e}_{2},$$

3 $$ M={i}_{3}+aX+{e}_{3}.$$

The simple relationship between the dependent variable (e.g., change in HIT-6 from baseline to 12-month) and the predictor, treatment (usual care vs. CHESS intervention), is represented by *c* (Fig. 1 (A)). The parameter *c’* represents the relationship between the dependent and predictor adjusted for the mediator (e.g., change in PSEQ from baseline to 4-month) whereas *b* represents the relationship between the dependent and mediator variables adjusted for the effects of the predictor (Fig. 1 (B)). The relationship between the predictor and mediator variable is represented by *a*. The mediated effect was the product of *a* and *b* parameters, *ab*. The 95% CI for the mediated effect was estimated with methods for asymmetric confidence limits.

All analyses were done with R (version 4.2.3) and the CI for the estimated effect was performed with the standard function *confint* and for the mediator effect, estimated with *medci* function (type = “prodclin”) from package RMediation [30, 31].

## Results

The CHESS trial randomised 736 participants, including for these exploratory analyses nine participants with chronic tension type headache that were not included in our primary analyses; 356 (48%) to usual care and 380 (52%) to self-management [14, 15]. Table 1 presents a brief summary of participants demographic and other characteristics at baseline.

**Table 1:** Demographics and characteristics at baseline

Characteristics	Treatment arms		
	Usual care (*N* = 356)	CHESS intervention (*N* = 380)	All (*N* = 736)
Age, years			
N	356	380	736
Mean (SD)	48 (15)	47 (15)	48 (15)
Median (IQR)	49 (37 to 58)	49 (36 to 57)	49 (36 to 58)
Gender, N (%)			
Male	71 (20)	55 (15)	126 (17.1)
Female	285 (80)	323 (85)	608 (82.6)
Missing	0 (0.0)	2 (1)	2 (< 1)
Types of headache, N (%)			
Chronic migraine	191 (54)	205 (54)	396 (54)
Chronic tension type headache plus episodic migraine	160 (45)	171 (45)	331 (45)
Chronic tension type headache	5 (1)	4 (1)	9 (1)
Medication overuse headache, N (%)			
Not medication overuse headache	158 (44)	168 (44)	326 (44)
Medication overuse headache	198 (56)	212 (56)	410 (56)
Number of days pain killers were used as acute medications for headache/migraine over the last 4 weeks			
N	350	375	725
Mean (SD)	12.3 (7.3)	12.5 (7.5)	12.4 (7.4)
Median (IQR)	12.0 (8.0 to 16.0)	12.0 (6.0 to 17.0)	12.0 (7.0 to 17.0)
Missing	6	5	11
HIT-6 (range, 36 to 78; higher is worse)			
N	355	378	733
Mean (SD)	64.4 (5.8)	64.3 (5.6)	64.4
Median (IQR)	64.0 (61.0 to 68.0)	64.0 (61.3 to 68.0)	64.0 (61.0 to 68.0)
Missing	1	2	3
PSEQ (range, 0 to 60; higher better)			
N	353	375	728
Mean (SD)	33.2 (13.4)	32.8 (13.8)	33.0 (13.6)
Median (IQR)	34.0 (24.0 to 43.0)	34.0 (22.5 to 44.0)	34.0 (23.8 to 44.0)
Missing	3	5	8
CH-QLQ Role Restrictive (range, 0 to 100; higher better)			
N	356	378	734
Mean (SD)	54.7 (17.6)	54.7 (17.1)	54.7
Median (IQR)	57.1 (42.9 to 66.7)	54.8 (42.9 to 66.7)	54.8 (42.9 to 66.7)
Missing	0	2	2
CH-QLQ Role Preventive (range, 0 to 100; higher better)			
N	356	378	734
Mean (SD)	69.7 (21.3)	69.7 (20.6)	69.7 (20.9)
Median (IQR)	75.0 (54.2 to 87.5)	70.8 (54.2 to 87.5)	70.8 (54.2 to 87.5)
Missing	0	2	2
CH-QLQ Emotional Function (range, 0 to 100; higher better)			
N	356	377	733
Mean (SD)	57.4 (22.4)	57.2 (22.4)	57.3 (22.4)
Median (IQR)	58.3 (38.9 to 77.8)	61.1 (38.9 to 77.8)	61.1 (38.9 to 77.8)
Missing	0	3	3

Abbreviations: SD, standard deviation; IQR, interquartile range, from 25th to 75th percentiles; HIT-6, Headache Specific Information; PSEQ, Pain Self-Efficacy Questionnaire; CH-QLQ, Chronic Headache Quality of Life Questionnaire

**Fig. 2:**
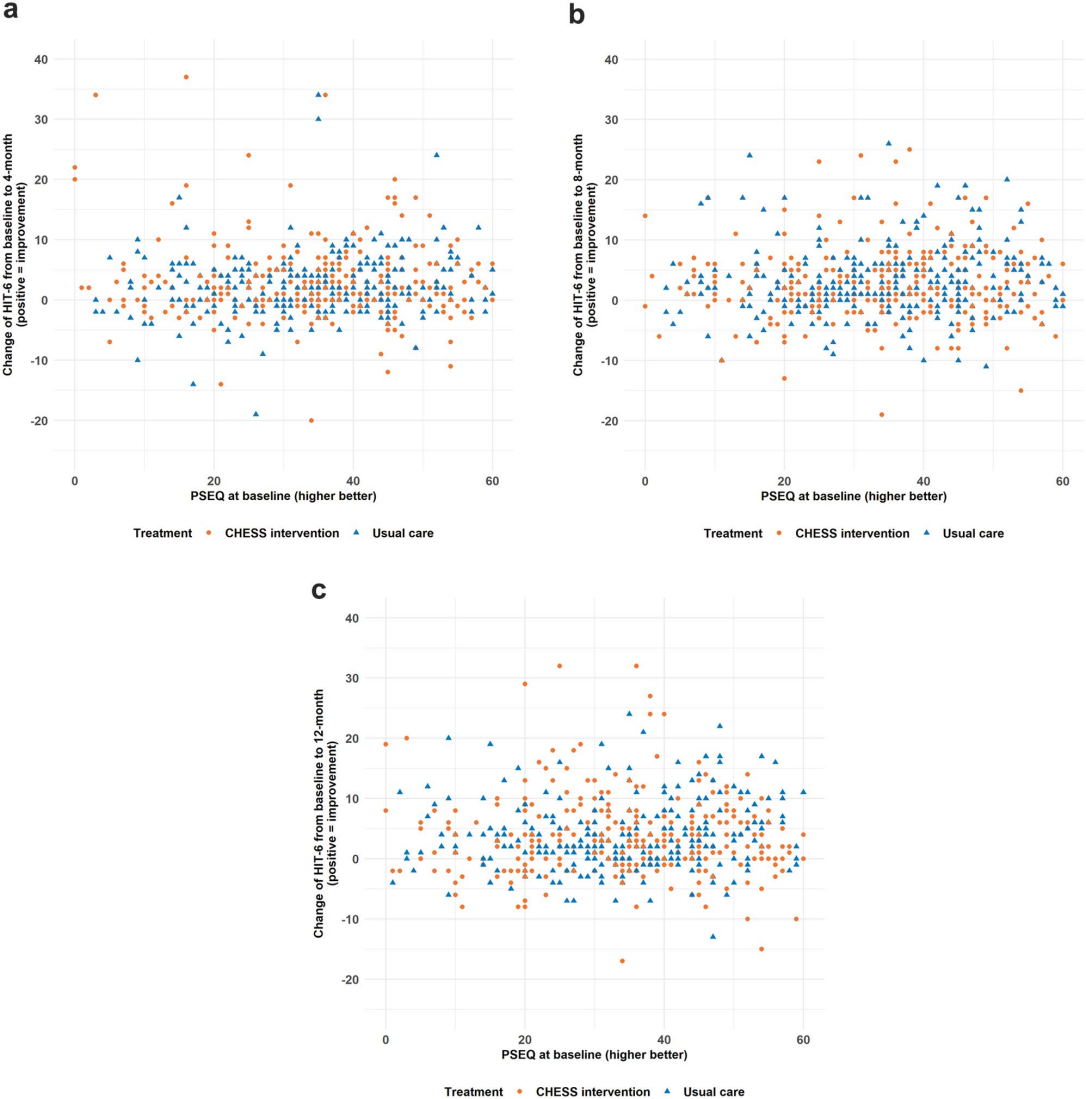
Change of Headache Specific Information (HIT-6) from baseline to (**a**) 4-month, (**b**) 8-month, and (**c**) 12-month by Pain Self-Efficacy Questionnaire (PSEQ) at baseline and by treatment with usual care denoted by blue triangle and CHESS intervention by orange circle

Figure 2 presents the scatter plot of change of HIT-6 from baseline to 4-, 8- and 12-month PSEQ at baseline by treatment arms. There was no obvious relationship between the change of HIT-6 and PSEQ at baseline and except that a small proportion of those reporting low PSEQ scores at baseline seemed to have greater improvement in HIT-6 at 4-month (Fig. 2a). Table 2 presents the estimated treatment and PSEQ at baseline effect with corresponding 95% CI. Treatment and PSEQ at baseline did not predict HIT-6 outcomes. There was a small ‘negative’ interaction effect of baseline PSEQ and treatment on HIT-6 at 4-month where participants with higher baseline PSEQ and randomised to the CHESS intervention seemed to report lower change of HIT-6 at 4-month.

**Table 2:** Estimated effect and its corresponding 95% confidence interval (CI) for predictor, moderator and mediator analyses for change of Headache Specific Information (HIT-6) from baseline to 4-, 8- and 12-month. The mediator is the change of Pain Self-Efficacy Questionnaire (PSEQ) from baseline to 4-month (*). Treatment usual care is the reference value. Scale range 36–78

Descriptions	Model/covariates	Estimated HIT-6 effect (95% CI)
Change from baseline to 4-month		
	***Predictors***	
	Treatment	0.815 (-0.170 to 1.799)
	PSEQ at baseline	0.009 (-0.028 to 0.046)
	***Moderators***	
	Treatment	4.129 (1.458 to 6.800)
	PSEQ at baseline	0.061 (0.007 to 0.114)
	Treatment × PSEQ at baseline	-0.098 (-0.172 to -0.025)
Change from baseline to 8-month		
	***Predictors***	
	Treatment	-0.256 (-1.259 to 0.747)
	PSEQ at baseline	0.015 (-0.023 to 0.052)
	***Moderators***	
	Treatment	1.073(-1.666 to 3.812)
	PSEQ at baseline	0.035 (-0.019 to 0.089)
	Treatment × PSEQ at baseline	-0.039 (-0.113 to 0.036)
	Mediator**	
	Direct treatment effect adjusted for mediator*, *c’*	-0.319 (-1.406 to 0.769)
	Mediated effect, *ab*	0.210 (0.033 to 0.453)
Change from baseline to 12-month		
	***Predictors***	
	Treatment	0.008 (-0.994 to 1.010)
	PSEQ at baseline	0.008 (-0.029 to 0.044)
	***Moderators***	
	Treatment	2.440 (-0.255 to 5.135)
	PSEQ at baseline	0.045 (-0.008 to 0.098)
	Treatment × PSEQ at baseline	-0.071 (-0.145 to 0.002)
	Mediator**	
	Direct treatment effect adjusted for mediator*, *c’*	-0.293 (-1.417 to 0.830)
	Mediated effect, *ab*	0.206 (0.031 to 0.451)

** The effect on the mediator (change of PSEQ from baseline to 4-month) from change of treatment, *a*, was 2.336 (95% CI, 0.484 to 4.187) for all outcomes. Note that the effect was not related to outcomes

The total effect of treatment on change of HIT-6 from baseline to 12-month (Fig. 1(A)) was 0.032 (*p* = 0.95, result not shown), indicating that there was no treatment effect on HIT-6. The CHESS intervention improved the mediated variable, PSEQ change from baseline to 4-month, by 2.34 (95% CI, 0.484 to 4.187) unit. This degree of change corresponds to an increase of 0.206 (95% CI, 0.031 to 0.451) unit in HIT-6 change from baseline to 12-month (Table 2) which was statistically significant as shown by the 95% CI which excludes zero. Tables 3, 4 and 5 present the estimated effects of treatment and baseline PSEQ baseline on CH-QLQ Role Restrictive, Role Preventive and Emotional Function outcomes. There was mediated effect on all outcomes with the greatest mediated effect was 1.12 (95% CI, 0.217 to 2.202) on CH-QLQ Emotional Function change from baseline to 12-month (Table 5). However, all the mediated effect seen were minimal, especially in CH-QLQ domain where the range is from 0 to 100. For completeness, in response to peer review and for the benefit of future systematic reviews, we present our analyses separately for participants with chronic migraine and those with chronic tension type headache plus episodic migraine in the Appendix. We draw no inference from these data.

**Table 3:** Estimated effect and its corresponding 95% confidence interval (CI) for predictor, moderator and mediator analyses for change of Chronic Headache Quality of Life Questionnaire (CH-QLQ) Role Restrictive from baseline to 4-, 8- and 12-month. The mediator is the change of Pain Self-Efficacy Questionnaire (PSEQ) from baseline to 4-month (*). Treatment usual care is the reference value. Scale range 0-100

Descriptions	Model/covariates	Estimated CH-QLQ Role Restrictive effect (95% CI)
Change from baseline to 4-month		
	***Predictors***	
	Treatment	1.242 (-1.441 to 3.924)
	PSEQ at baseline	-0.091 (-0.193 to 0.010)
	***Moderators***	
	Treatment	1.933 (-5.548 to 9.414)
	PSEQ at baseline	-0.081 (-0.229 to 0.068)
	Treatment × PSEQ at baseline	-0.020 (-0.223 to 0.183)
Change from baseline to 8-month		
	***Predictors***	
	Treatment	-0.392 (-3.325 to 2.540)
	PSEQ at baseline	-0.113 (-0.222 to -0.004)
	***Moderators***	
	Treatment	0.392 (-7.723 to 8.507)
	PSEQ at baseline	-0.101 (-0.262 to 0.060)
	Treatment × PSEQ at baseline	-0.023 (-0.241 to 0.196)
	Mediator**	
	Direct treatment effect adjusted for mediator*, *c’*	-1.422 (-4.447 to 1.604)
	Mediated effect, *ab*	0.793 (0.149 to 1.600)
Change from baseline to 12-month		
	***Predictors***	
	Treatment	0.151 (-2.712 to 3.014)
	PSEQ at baseline	-0.073 (-0.178 to 0.032)
	***Moderators***	
	Treatment	1.093 (-6.713 to 8.900)
	PSEQ at baseline	-0.058 (-0.213 to 0.097)
	Treatment × PSEQ at baseline	-0.027 (-0.238 to 0.183)
	Mediator**	
	Direct treatment effect adjusted for mediator*, *c’*	-0.816 (-3.767 to 2.134)
	Mediated effect, *ab*	0.831 (0.160 to 1.655)

** The effect on the mediator (change of PSEQ from baseline to 4-month) from change of treatment, *a*, was 2.336 (95% CI, 0.484 to 4.187) for all outcomes. Note that the effect was not related to outcomes

**Table 4:** Estimated effect and its corresponding 95% confidence interval (CI) for predictor, moderator and mediator analyses for change of Chronic Headache Quality of Life Questionnaire (CH-QLQ) Role Preventive from baseline to 4-, 8- and 12-month. The mediator is the change of Pain Self-Efficacy Questionnaire (PSEQ) from baseline to 4-month (*). Treatment usual care is the reference value. Scale range 0-100

Descriptions	Model/covariates	Estimated CH-QLQ Role Preventive effect (95% CI)
Change from baseline to 4-month		
	***Predictors***	
	Treatment	1.758 (-0.930 to 4.446)
	PSEQ at baseline	-0.200 (-0.301 to -0.099)
	***Moderators***	
	Treatment	4.102 (-3.393 to 11.596)
	PSEQ at baseline	-0.164 (-0.312 to -0.015)
	Treatment × PSEQ at baseline	-0.068 (-0.271 to 0.135)
Change from baseline to 8-month		
	***Predictors***	
	Treatment	1.080 (-1.739 to 3.898)
	PSEQ at baseline	-0.202 (-0.307 to -0.097)
	***Moderators***	
	Treatment	2.873 (-4.926 to 10.673)
	PSEQ at baseline	-0.174 (-0.328 to -0.020)
	Treatment × PSEQ at baseline	-0.052 (-0.262 to 0.158)
	Mediator**	
	Direct treatment effect adjusted for mediator*, *c’*	-0.253 (-3.206 to 2.701)
	Mediated effect, *ab*	0.769 (0.145 to 1.554)
Change from baseline to 12-month		
	***Predictors***	
	Treatment	2.531 (-0.471 to 5.533)
	PSEQ at baseline	-0.2093 (-0.319 to -0.099)
	***Moderators***	
	Treatment	5.848 (-2.332 to 14.027)
	PSEQ at baseline	-0.157 (-0.319 to 0.005)
	Treatment × PSEQ at baseline	-0.096 (-0.317 to 0.124)
	Mediator**	
	Direct treatment effect adjusted for mediator*, *c’*	1.616 (-1.65 to 4.877)
	Mediated effect, *ab*	0.758 (0.137 to 1.564)

** The effect on the mediator (change of PSEQ from baseline to 4-month) from change of treatment, *a*, was 2.336 (95% CI, 0.484 to 4.187) for all outcomes. Note that the effect was not related to outcomes

**Table 5:** Estimated effect and its corresponding 95% confidence interval (CI) for predictor, moderator and mediator analyses for change of Chronic Headache Quality of Life Questionnaire (CH-QLQ) Emotional Function from baseline to 4-, 8- and 12-month. The mediator is the change of Pain Self-Efficacy Questionnaire (PSEQ) from baseline to 4-month (*). Treatment usual care is the reference value. Scale range 0-100

Descriptions	Model/covariates	Estimated CH-QLQ Emotional Function effect (95% CI)
Change from baseline to 4-month		
	***Predictors***	
	Treatment	1.380 (-1.936 to 4.697)
	PSEQ at baseline	-0.074 (-0.199 to 0.051)
	***Moderators***	
	Treatment	1.491 (-7.760 to 10.741)
	PSEQ at baseline	-0.072 (-0.255 to 0.111)
	Treatment × PSEQ at baseline	-0.003 (-0.254 to 0.248)
Change from baseline to 8-month		
	***Predictors***	
	Treatment	-0.615 (-4.203 to 2.974)
	PSEQ at baseline	-0.1915 (-0.325 to -0.058)
	***Moderators***	
	Treatment	7.714 (-2.176 to 17.605)
	PSEQ at baseline	-0.062 (-0.257 to 0.134)
	Treatment × PSEQ at baseline	-0.241 (-0.507 to 0.026)
	Mediator**	
	Direct treatment effect adjusted for mediator*, *c’*	-0.974 (-4.714 to 2.766)
	Mediated effect, *ab*	0.864 (0.155 to 1.786)
Change from baseline to 12-month		
	***Predictors***	
	Treatment	1.241 (-2.351 to 4.832)
	PSEQ at baseline	-0.133 (-0.265 to -0.001)
	***Moderators***	
	Treatment	4.644 (-5.165 to 14.454)
	PSEQ at baseline	-0.080 (-0.274 to 0.114)
	Treatment × PSEQ at baseline	-0.099 (-0.363 to 0.166)
	Mediator**	
	Direct treatment effect adjusted for mediator*, *c’*	0.433 (-3.309 to 4.174)
	Mediated effect, *ab*	1.116 (0.217 to 2.202)

** The effect on the mediator (change of PSEQ from baseline to 4-month) from change of treatment, *a*, was 2.336 (95% CI, 0.484 to 4.187) for all outcomes. Note that the effect was not related to outcomes

## Discussion

In our analysis of all 736 randomised participants, we found that the CHESS intervention improved scores four-month PSEQ by 2.3 points (95% CI, 0.48 to 4.19) on a 60-point scale when compared to usual care. What might be a worthwhile difference on PSEQ in this context has not been established. In a study of people with low back pain the minimally important within person change in PSEQ was found to be 5.5 [25]. Using a benchmark of half of the minimally important within person change to indicate a worthwhile benefit this might indicate that the CHESS intervention has not had a meaningful impact on PSEQ [26]. This is reflected in the findings of our mediation analyses. For the HIT-6, the primary outcome for the CHESS trial, the impact of the observed changes in PSEQ, across both groups, was 0.21 (95% CI, 0.03 to 0.45). This is just over 10% of the target difference of 2.0 points on the HIT-6 set for the CHESS trial [17]. Worthwhile differences on the CH-QLQ domains have not been established. Nevertheless, even the largest observed difference of 1.12 (95% CI, 0.22 to 2.20) on emotional function at one year, measured on a 0-100 scale is very unlikely to be clinically important. Thus, we have shown that we can influence pain self-efficacy, and that changes in self-efficacy mediate clinical outcomes. However, these effects are small, or even trivial, and unlikely to be of clinical importance.

Our process evaluation of the CHESS trial did not indicate why our intervention was ineffective [18]. These new analyses perhaps give some insight into why this might be. A key target of the CHESS intervention was improving self-efficacy [22]. That improved self-efficacy does mediate clinical outcomes indicates that this was an appropriate target. Our intervention did not have a large enough effect on self-efficacy to be clinically useful. Future studies in this area may need to consider how to maximise any effect on self-efficacy. This may be challenging. A 2020 systematic review of 60 randomised controlled trials in people with chronic musculoskeletal pain found that exercise interventions and multicomponent interventions had small effects on self-efficacy at 4–6 months; standardised mean differences of 0.33 and 0.27, respectively. But no effect was seen from self-management interventions or psychological therapies [27]. We are not aware of similar data from studies of headache disorders. Our observed standardised mean difference of 0.17 is even smaller indicating that a much more intense and/or lengthier intervention would be needed to have a clinically worthwhile effect. An alternative perspective would be to accept that self-efficacy has such a small effect, with little prospect of achieving sufficient change to have a worthwhile clinical effect that it is not worth pursuing this line of enquiry further. Except, perhaps as part of a wider multicomponent intervention where self-efficacy is one of a range of targets.

### Strengths and weaknesses

The CHESS trial is one of the largest studies of non-drug interventions for chronic headache disorders providing a good dataset to test the hypothesis that changes in self-efficacy might mediate improvements in later clinical outcomes. However, what we reported here are post-hoc secondary analyses not specified in the original statistical analysis plan. Some caution is therefore needed when interpreting these post-hoc analyses. We have done multiple analyses, meaning some of the observed statistically significant mediation effects observed might be random chance. That across all the outcomes measured a consistent pattern of mediation is observed at 12 months gives some reassurance that this is not the case. We pooled the three subtypes of chronic headaches (namely, chronic migraine, chronic tension type headache with episodic migraine and chronic tension type headache) in our exploratory analyses to reduce the likelihood of observing significant effect by random chance. Similarly, we have based this analysis on our primary outcome and not tested if changes in self-efficacy mediate change on the CH-QLQ. This may, however, be a more relevant outcome for future studies in people with chronic headaches. The Migraine Functional Impact Questionnaire may be a more relevant outcome for studies of people with chronic migraine [28, 29]. 

## Conclusion

The CHESS intervention has positive effect on self-efficacy at four months. This change in self-efficacy mediates an improvement in HIT-6 scores at 12 months, but is very minimal. Future behavioural interventions for people with chronic migraine need to consider how to maximise the effect on self-efficacy and to consider which other factors could be targeted. It is likely a much more intense intervention than that tested within the CHESS trial will be needed.

### Electronic supplementary material

Below is the link to the electronic supplementary material.

Supplementary Material 1

## Data Availability

The data that support the findings of this study are available from Warwick Clinical Trials Unit (wctudataaccess@warwick.ac.uk) but restrictions apply to the availability of these data, which were used under license for the current study, and so are not publicly available. Data are however available from the authors upon reasonable request and with permission of Warwick Clinical Trials Unit.

## Abbreviations

CH-QLQ: Chronic headache quality of life questionnaire
CHESS: Chronic Headache Education and Self-management Study
CI: Confidence interval
HIT-6: Headache Impact Test − 6
NIHR: National Institute for Health Research
PSEQ: Pain self-efficacy questionnaire

## References

[1] Naghdi S, Underwood M, Madan J, Brown A, Duncan C, Matharu M (2023). Clinical effectiveness of pharmacological interventions for managing chronic migraine in adults: a systematic review and network meta-analysis. J Headache Pain.

[2] Sharpe L, Dudeney J, Williams ACC, Nicholas M, McPhee I, Baillie A (2019). Psychological therapies for the prevention of migraine in adults. Cochrane Database Syst Rev.

[3] Kristoffersen ES, Lundqvist C, Russell MB (2019). Illness perception in people with primary and secondary chronic headache in the general population. J Psychosom Res.

[4] Westergaard ML, Lau CJ, Allesoe K, Gjendal ST, Jensen RH (2020). Monitoring chronic headache and medication-overuse headache prevalence in Denmark. Cephalalgia.

[5] Henning V, Katsarava Z, Obermann M, Moebus S, Schramm S (2018). Remission of chronic headache: Rates, potential predictors and the role of medication, follow-up results of the German Headache Consortium (GHC) Study. Cephalalgia.

[6] Nichols VP, Ellard DR, Griffiths FE, Kamal A, Underwood M, Taylor SJC (2017). The lived experience of chronic headache: a systematic review and synthesis of the qualitative literature. BMJ Open.

[7] Battista S, Lazzaretti A, Coppola I, Falsiroli Maistrello L, Rania N, Testa M (2023). Living with migraine: a meta-synthesis of qualitative studies. Front Psychol.

[8] Scaratti C, Covelli V, Guastafierro E, Leonardi M, Grazzi L, Rizzoli PB (2018). A qualitative study on patients with chronic migraine with medication overuse headache: comparing frequent and non-frequent relapsers. Headache.

[9] Palacios-Cena D, Neira-Martin B, Silva-Hernandez L, Mayo-Canalejo D, Florencio LL, Fernandez-de-Las-Penas C (2017). Living with chronic migraine: a qualitative study on female patients’ perspectives from a specialised headache clinic in Spain. BMJ Open.

[10] Vivekanantham A, Edwin C, Pincus T, Matharu M, Parsons H, Underwood M (2019). The association between headache and low back pain: a systematic review. J Headache Pain.

[11] Probyn K, Bowers H, Caldwell F, Mistry D, Underwood M, Matharu M (2017). Prognostic factors for chronic headache: a systematic review. Neurology.

[12] Bandura A (1977). Self-efficacy: toward a unifying theory of behavioral change. Psychol Rev.

[13] Henningsen P, Hausteiner-Wiehle C, Hauser W (2022). Migraine in the context of chronic primary pain, chronic overlapping pain disorders, and functional somatic disorders: a narrative review. Headache.

[14] Underwood M, Achana F, Carnes D, Eldridge S, Ellard DR, Griffiths F et al Non-pharmacological educational and self-management interventions for people with chronic headache: the CHESS research programme including a RCT. Programme Grants for Applied Research. Southampton (UK)2023. 37463270

[15] Underwood M, Achana F, Carnes D, Eldridge S, Ellard DR, Griffiths F (2023). Supportive self-management program for people with chronic headaches and migraine: a randomized controlled trial and economic evaluation. Neurology.

[16] Kosinski M, Bayliss MS, Bjorner JB, Ware JE, Garber WH, Batenhorst A (2003). A six-item short-form survey for measuring headache impact: the HIT-6. Qual Life Res.

[17] Patel S, Achana F, Carnes D, Eldridge S, Ellard DR, Griffiths F (2020). Usual care and a self-management support programme versus usual care and a relaxation programme for people living with chronic headache disorders: a randomised controlled trial protocol (CHESS). BMJ Open.

[18] Ellard DR, Nichols VP, Griffiths FE, Underwood M, Taylor SJC (2023) team C. Chronic Headache Education and Self-Management Study (CHESS): a process evaluation. BMC Neurol.;23(1):8 10.1186/s12883-022-02792-1 PMC9823254 36609224

[19] Haywood KL, Mars TS, Potter R, Patel S, Matharu M, Underwood M (2018). Assessing the impact of headaches and the outcomes of treatment: a systematic review of patient-reported outcome measures (PROMs). Cephalalgia.

[20] Haywood KL, Achana F, Nichols V, Pearce G, Box B, Muldoon L (2021). Measuring health-related quality of life in chronic headache: a comparative evaluation of the chronic Headache Quality of Life Questionnaire and Headache Impact Test (HIT-6). Cephalalgia.

[21] White K, Potter R, Patel S, Nichols VP, Haywood KL, Hee SW (2019). Chronic Headache Education and Self-management study (CHESS) - a mixed method feasibility study to inform the design of a randomised controlled trial. BMC Med Res Methodol.

[22] Patel S, Potter R, Matharu M, Carnes D, Taylor SJC, Nichols V (2019). Development of an education and self-management intervention for chronic headache - CHESS trial (chronic Headache Education and Self-management study). J Headache Pain.

[23] Nicholas MK (2007). The pain self-efficacy questionnaire: taking pain into account. Eur J Pain.

[24] Potter R, Hee SW, Griffiths F, Dodd K, Hoverd E, Underwood M (2019). Development and validation of a telephone classification interview for common chronic headache disorders. J Headache Pain.

[25] Chiarotto A, Vanti C, Cedraschi C, Ferrari S, de Lima ESRF, Ostelo RW (2016). Responsiveness and minimal important change of the Pain Self-Efficacy Questionnaire and short forms in patients with chronic low back Pain. J Pain.

[26] Johnston BC, Thorlund K, Schunemann HJ, Xie F, Murad MH, Montori VM (2010). Improving the interpretation of quality of life evidence in meta-analyses: the application of minimal important difference units. Health Qual Life Outcomes.

[27] Martinez-Calderon J, Flores-Cortes M, Morales-Asencio JM, Fernandez-Sanchez M, Luque-Suarez A (2020). Which interventions enhance Pain Self-efficacy in people with Chronic Musculoskeletal Pain? A systematic review with Meta-analysis of Randomized controlled trials, including over 12 000 participants. J Orthop Sports Phys Ther.

[28] Hareendran A, Skalicky A, Mannix S, Lavoie S, Desai P, Bayliss M (2018). Development of a New Tool for evaluating the benefit of preventive treatments for migraine on functional outcomes - the Migraine Functional Impact Questionnaire (MFIQ). Headache.

[29] Haywood K, Potter R, Froud R, Pearce G, Box B, Muldoon L (2021). Core outcome set for preventive intervention trials in chronic and episodic migraine (COSMIG): an international, consensus-derived and multistakeholder initiative. BMJ Open.

[30] R Core Team (2023) R: A language and environment for statistical computing. R Foundation for Statistical Computing, Vienna, Austria. https://www.R-project.org/

[31] Tofighi D, MacKinnon DP (2011) RMediation: An R package for mediation analysis confidence intervals. Behav Res 43, 692–700. 10.3758/s13428-011-0076-x 10.3758/s13428-011-0076-x PMC3233842 21487904

