# Supplementary material for: Does pain self-efficacy predict, moderate or mediate outcomes in people with chronic headache; an exploratory analysis of the CHESS trial
Source: J Headache Pain. 2024 May 15;25(1):77. doi: 10.1186/s10194-024-01768-5 (PMC11094916; doi:10.1186/s10194-024-01768-5)
Supplement: Supplementary file 1 — Supplementary Material 1 [file 10194_2024_1768_MOESM1_ESM.pdf]

Does pain self-efficacy predict, moderate or mediate outcomes in people with chronic headache; an exploratory analysis of the CHESS trial

Siew Wan Hee, Shilpa Patel, Harbdiner Sandhu, Manjit S. Matharu and Martin Underwood on behalf of the CHESS Team

Appendix 1 Chronic migraine sub-group analysis

Table A1.1 Demographics and characteristics at baseline.

Characteristics	Treatment arms: Usual care (N = 191)	Treatment arms: CHESS intervention (N = 205)	All (N = 396)
Age, years			
N	191	205	396
Mean (SD)	47.6 (13.9)	46.4 (14.8)	47.0 (14.4)
Median (IQR)	49.0 (37.0 to 58.0)	48.0 (36.0 to 56.0)	49.0 (36.0 to 57.0)
Gender, N (%)			
Male	31 (16.2)	30 (14.6)	61 (15.4)
Female	160 (83.8)	173 (84.4)	333 (84.1)
Missing	0 (0.0)	2 (1.0)	2 (0.5)
Medication overuse headache, N (%)			
Not medication overuse headache	69 (36.1)	74 (36.1)	143 (36.1)
Medication overuse headache	122 (63.9)	131 (63.9)	253 (63.9)
Number of days pain killers were used as acute medications for headache/migraine over the last 4 weeks			
N	187	201	388
Mean (SD)	12.9 (6.8)	13.1 (7.6)	13.0 (7.2)
Median (IQR)	12.0 (9.0 to 16.0)	14.0 (7.0 to 18.0)	12.0 (8.0 to 18.0)
Missing	4	4	8
HIT-6 (range, 36 to 78; higher is worse)			
N	190	203	393
Mean (SD)	66.5 (4.9)	65.8 (4.9)	66.1 (4.9)
Median (IQR)	66.0 (63.0 to 70.0)	66.0 (63.0 to 69.0)	66.0 (63.0 to 69.0)
Missing	1	2	3
PSEQ (range, 0 to 60; higher better)			
N	190	202	392
Mean (SD)	29.6 (13.4)	30.3 (13.8)	30.0 (13.6)
Median (IQR)	30.0 (19.0 to 39.8)	31.0 (21.0 to 39.8)	30.0 (20.0 to 40.0)
Missing	1	3	4
CH-QLQ Role Restrictive (range, 0 to 100; higher better)			
N	191	203	394
Mean (SD)	49.6 (16.3)	49.7 (16.6)	49.7 (16.4)
Median (IQR)	50.0 (38.1 to 61.9)	52.4 (36.9 to 59.5)	50.0 (38.1 to 61.3)
Missing	0	2	2

Characteristics	Treatment arms: Usual care (N = 191)	Treatment arms: CHESS intervention (N = 205)	All (N = 396)
CH-QLQ Role Preventive (range, 0 to 100; higher better)			
N	191	203	394
Mean (SD)	63.1 (21.5)	64.8 (20.8)	63.9 (21.2)
Median (IQR)	66.7 (45.8 to 79.2)	66.7 (50.0 to 79.2)	66.7 (50.0 to 79.2)
Missing	0	2	2
CH-QLQ Emotional Function (range, 0 to 100; higher better)			
N	191	202	393
Mean (SD)	52.3 (22.3)	51.5 (21.3)	51.9 (21.8)
Median (IQR)	50.0 (33.3 to 72.2)	50.0 (33.3 to 70.8)	50.0 (33.3 to 72.2)
Missing	0	3	3

Abbreviations: SD, standard deviation; IQR, interquartile range, from 25th to 75th percentiles; HIT-6, Headache Specific Information; PSEQ, Pain Self-Efficacy Questionnaire; CH-QLQ, Chronic Headache Quality of Life Questionnaire.

Table A1.2 Estimated effect and its corresponding 95% confidence interval (CI) for predictor, moderator and mediator analyses for change of Headache Specific Information (HIT-6), Chronic Headache Quality of Life Questionnaire (CH-QLQ) Role Restrictive, CH-QLQ Role Preventive and CH-QLQ Emotional Function from baseline to 4-, 8- and 12-month. The mediator is the change of Pain Self-Efficacy Questionnaire (PSEQ) from baseline to 4-month (*). Treatment usual care is the reference value and positive change indicates improvement.

		Estimated value (95% CI)			
**Descriptions**	**Model/covariates**	**HIT-6 (range, 36 to 78; higher is worse)**	**CH-QLQ Role Restrictive (range, 0 to 100; higher better)**	**CH-QLQ Role Preventive effect (range, 0 to 100; higher better)**	**CH-QLQ Emotional Function (range, 0 to 100; higher better)**
Change from baseline to 4-month					
	Predictors				
	Treatment	0.892 (−0.288 to 2.071)	1.794 (−2.232 to 5.821)	1.397 (−2.542 to 5.336)	2.651 (−2.127 to 7.429)
	PSEQ at baseline	0.029 (−0.015 to 0.074)	−0.043 (−0.194 to 0.108)	−0.136 (−0.283 to 0.012)	0.006 (−0.173 to 0.185)
	Moderators				
	Treatment	2.219 (−0.796 to 5.234)	−1.471 (−11.845 to 8.904)	3.112 (−7.044 to 13.269)	−3.707 (−16 to 8.585)
	PSEQ at baseline	0.051 (−0.013 to 0.114)	−0.097 (−0.317 to 0.122)	−0.107 (−0.322 to 0.108)	−0.1 (−0.36 to 0.16)
	Treatment × PSEQ at baseline	−0.043 (−0.132 to 0.047)	0.103 (−0.199 to 0.406)	−0.054 (−0.351 to 0.242)	0.201 (−0.157 to 0.56)
Change from baseline to 8-month					
	Predictors				
	Treatment	−0.026 (−1.415 to 1.363)	0.673 (−3.863 to 5.209)	0.754 (−3.543 to 5.05)	1.52 (−3.974 to 7.014)
	PSEQ at baseline	0.001 (−0.051 to 0.053)	−0.106 (−0.274 to 0.063)	−0.166 (−0.325 to −0.006)	−0.121 (−0.324 to 0.082)
	Moderators				
	Treatment	0.960 (−2.568 to 4.487)	−0.599 (−12.143 to 10.944)	0.925 (−10.01 to 11.86)	2.718 (−11.243 to 16.679)
	PSEQ at baseline	0.017 (−0.057 to 0.092)	−0.128 (−0.379 to 0.123)	−0.163 (−0.401 to 0.075)	−0.1 (−0.404 to 0.204)
	Treatment × PSEQ at baseline	−0.032 (−0.135 to 0.072)	0.041 (−0.298 to 0.379)	−0.005 (−0.326 to 0.315)	−0.038 (−0.448 to 0.371)
	Mediator*				
	Direct treatment effect adjusted for mediator*, *c'*	−0.068 (−1.637 to 1.501)	−0.733 (−5.515 to 4.049)	−0.943 (−5.509 to 3.623)	1.321 (−4.43 to 7.072)
	Mediated effect, *ab*	0.199 (−0.0235 to 0.557)	0.793 (−0.006 to 2.008)	0.746 (−0.009 to 1.899)	0.657 (−0.127 to 1.918)

Chronic migraine sub-group analysis

		Estimated value (95% CI)			
**Descriptions**	**Model/covariates**	**HIT-6 (range, 36 to 78; higher is worse)**	**CH-QLQ Role Restrictive (range, 0 to 100; higher better)**	**CH-QLQ Role Preventive effect (range, 0 to 100; higher better)**	**CH-QLQ Emotional Function (range, 0 to 100; higher better)**
Change from baseline to 12-month					
	Predictors				
	Treatment	0.237 (−1.170 to 1.645)	0.973 (−3.243 to 5.189)	2.998 (−1.612 to 7.607)	2.504 (−2.659 to 7.667)
	PSEQ at baseline	−0.013 (−0.064 to 0.039)	−0.136 (−0.29 to 0.018)	−0.175 (−0.344 to −0.007)	−0.171 (−0.36 to 0.018)
	Moderators				
	Treatment	2.185 (−1.314 to 5.684)	1.406 (−9.154 to 11.966)	5.109 (−6.432 to 16.65)	2.129 (−10.848 to 15.106)
	PSEQ at baseline	0.020 (−0.055 to 0.095)	−0.129 (−0.358 to 0.101)	−0.138 (−0.389 to 0.113)	−0.178 (−0.458 to 0.103)
	Treatment × PSEQ at baseline	−0.063 (−0.166 to 0.040)	−0.014 (−0.324 to 0.297)	−0.068 (−0.407 to 0.272)	0.012 (−0.369 to 0.393)
	Mediator				
	Direct treatment effect adjusted for mediator*, *c'*	0.397 (−1.241 to 2.034)	0.764 (−3.79 to 5.317)	2.501 (−2.627 to 7.628)	2.337 (−3.1 to 7.774)
	Mediated effect, *ab*	0.316 (0.006 to 0.765)	0.836 (0.009 to 2.052)	0.651 (−0.077 to 1.82)	1.316 (0.048 to 3.006)

** The effect on the mediator (change of PSEQ from baseline to 4-month) from change of treatment, *a*, was 2.696 (0.116 to 5.275) for all outcomes from baseline to 8- and 12-month.

Chronic migraine sub-group analysis

Appendix 2 Chronic tension type headache plus episodic migraine sub-group analysis

Table A2.1 Demographics and characteristics at baseline.

Characteristics	Treatment arms: Usual care (N = 160)	Treatment arms: CHESS intervention (N = 171)	All (N = 331)
Age, years			
N	160	171	331
Mean (SD)	48.3 (16.2)	47.8 (15.1)	48.1 (15.6)
Median (IQR)	48 (36.8 to 59.2)	50 (35.0 to 58.0)	49 (36.0 to 59.0)
Gender, N (%)			
Male	31 (16.2%)	30 (14.6%)	61 (15.4%)
Female	160 (83.8%)	173 (84.4%)	333 (84.1%)
Missing	0 (0.0%)	2 (1.0%)	2 (0.5%)
Medication overuse headache, N (%)			
Not medication overuse headache	69 (36.1%)	74 (36.1%)	143 (36.1%)
Medication overuse headache	122 (63.9%)	131 (63.9%)	253 (63.9%)
Number of days pain killers were used as acute medications for headache/migraine over the last 4 weeks			
N	159	170	329
Mean (SD)	11.8 (7.8)	11.8 (7.3)	11.8 (7.5)
Median (IQR)	10 (6.0 to 16.0)	10.5 (6.0 to 15.0)	10 (6.0 to 16.0)
Missing	1	1	2
HIT-6 (range, 36 to 78; higher is worse)			
N	160	171	331
Mean (SD)	62.3 (5.4)	62.8 (5.7)	62.6 (5.5)
Median (IQR)	63 (59.0 to 66.0)	63 (60.0 to 66.0)	63 (60.0 to 66.0)
Missing	0	0	0
PSEQ (range, 0 to 60; higher better)			
N	158	169	327
Mean (SD)	36.9 (12.1)	35.2 (13.2)	36.0 (12.7)
Median (IQR)	37 (28.0 to 45.0)	36 (27.0 to 45.0)	37 (27.0 to 45.0)
Missing	2	2	4
CH-QLQ Role Restrictive (range, 0 to 100; higher better)			
N	160	171	331
Mean (SD)	60.3 (16.7)	60.0 (15.6)	60.1 (16.1)
Median (IQR)	61.9 (50.0 to 71.4)	61.9 (50.0 to 69.0)	61.9 (50.0 to 71.4)
Missing	0	0	0
CH-QLQ Role Preventive (range, 0 to 100; higher better)			
N	160	171	331
Mean (SD)	77.0 (18.0)	75.0 (18.7)	76.0 (18.4)
Median (IQR)	83.3 (66.7 to 91.7)	79.2 (62.5 to 91.7)	79.2 (66.7 to 91.7)
Missing	0	0	0

Characteristics	Treatment arms: Usual care (N = 160)	Treatment arms: CHESS intervention (N = 171)	All (N = 331)
CH-QLQ Emotional Function (range, 0 to 100; higher better)			
N	160	171	331
Mean (SD)	63.1 (20.9)	63.5 (21.9)	63.3 (21.4)
Median (IQR)	66.7 (50.0 to 83.3)	66.7 (44.4 to 83.3)	66.7 (47.2 to 83.3)
Missing	0	0	0

Abbreviations: SD, standard deviation; IQR, interquartile range, from 25th to 75th percentiles; HIT-6, Headache Specific Information; PSEQ, Pain Self-Efficacy Questionnaire; CH-QLQ, Chronic Headache Quality of Life Questionnaire.

Chronic tension type headache plus episodic migraine sub-group analysis

Table A2.2 Estimated effect and its corresponding 95% confidence interval (CI) for predictor, moderator and mediator analyses for change of Headache Specific Information (HIT-6), Chronic Headache Quality of Life Questionnaire (CH-QLQ) Role Restrictive, CH-QLQ Role Preventive and CH-QLQ Emotional Function from baseline to 4-, 8- and 12-month. The mediator is the change of Pain Self-Efficacy Questionnaire (PSEQ) from baseline to 4-month (*). Treatment usual care is the reference value and positive change indicates improvement.

		Estimated value (95% CI)			
**Descriptions**	**Model/covariates**	**HIT-6 (range, 36 to 78; higher is worse)**	**CH-QLQ Role Restrictive (range, 0 to 100; higher better)**	**CH-QLQ Role Preventive effect (range, 0 to 100; higher better)**	**CH-QLQ Emotional Function (range, 0 to 100; higher better)**
Change from baseline to 4-month					
	Predictors				
	Treatment	0.719 (−0.933 to 2.371)	0.414 (−3.185 to 4.013)	1.75 (−1.96 to 5.46)	−0.939 (−5.588 to 3.71)
	PSEQ at baseline	−0.021 (−0.086 to 0.043)	−0.149 (−0.294 to −0.004)	−0.316 (−0.466 to −0.167)	−0.202 (−0.389 to −0.014)
	Moderators				
	Treatment	6.653 (1.621 to 11.684)	5.825 (−5.651 to 17.301)	4.159 (−7.692 to 16.009)	6.852 (−7.965 to 21.669)
	PSEQ at baseline	0.073 (−0.026 to 0.173)	−0.066 (−0.288 to 0.155)	−0.279 (−0.508 to −0.051)	−0.083 (−0.368 to 0.203)
	Treatment × PSEQ at baseline	−0.162 (−0.293 to −0.032)	−0.146 (−0.439 to 0.148)	−0.065 (−0.367 to 0.238)	−0.21 (−0.588 to 0.169)
Change from baseline to 8−month					
	Predictors				
	Treatment	−0.321 (−1.803 to 1.161)	−1.717 (−5.507 to 2.073)	1.129 (−2.61 to 4.868)	−3.629 (−8.298 to 1.04)
	PSEQ at baseline	0.043 (−0.015 to 0.101)	−0.109 (−0.259 to 0.041)	−0.249 (−0.397 to −0.101)	−0.267 (−0.451 to −0.082)
	Moderators				
	Treatment	1.760 (−2.869 to 6.388)	1.102 (−10.971 to 13.175)	5.71 (−6.19 to 17.61)	13.941 (−0.744 to 28.626)
	PSEQ at baseline	0.075 (−0.014 to 0.164)	−0.066 (−0.296 to 0.164)	−0.179 (−0.406 to 0.047)	0 (−0.28 to 0.28)
	Treatment × PSEQ at baseline	−0.056 (−0.173 to 0.062)	−0.075 (−0.379 to 0.229)	−0.121 (−0.421 to 0.178)	−0.466 (−0.835 to −0.096)
	Mediator*				
	Direct treatment effect adjusted for mediator*, *c'*	−0.517 (−2.056 to 1.021)	−2.21 (−5.993 to 1.572)	0.414 (−3.427 to 4.254)	−3.593 (−8.425 to 1.239)
	Mediated effect, *ab*	0.179 (−0.102 to 0.553)	0.627 (−0.357 to 1.801)	0.64 (−0.365 to 1.837)	0.796 (−0.453 to 2.289)

Chronic tension type headache plus episodic migraine sub-group analysis

Descriptions	Model/covariates	Estimated value (95% CI)			
		**HIT-6 (range, 36 to 78; higher is worse)**	**CH-QLQ Role Restrictive (range, 0 to 100; higher better)**	**CH-QLQ Role Preventive effect (range, 0 to 100; higher better)**	**CH-QLQ Emotional Function (range, 0 to 100; higher better)**
Change from baseline to 12−month					
	Predictors				
	Treatment	−0.088 (−1.556 to 1.381)	−0.505 (−4.505 to 3.495)	1.863 (−2.128 to 5.854)	−0.346 (−5.515 to 4.824)
	PSEQ at baseline	0.042 (−0.016 to 0.099)	−0.018 (−0.174 to 0.138)	−0.267 (−0.423 to −0.111)	−0.105 (−0.307 to 0.097)
	Moderators				
	Treatment	3.368 (−1.158 to 7.894)	−0.455 (−13.078 to 12.168)	5.331 (−7.254 to 17.917)	7.622 (−8.657 to 23.901)
	PSEQ at baseline	0.094 (0.008 to 0.180)	−0.017 (−0.26 to 0.225)	−0.213 (−0.455 to 0.029)	0.019 (−0.294 to 0.332)
	Treatment × PSEQ at baseline	−0.093 (−0.208 to 0.022)	−0.001 (−0.319 to 0.316)	−0.092 (−0.409 to 0.225)	−0.212 (−0.621 to 0.198)
	Mediator**				
	Direct treatment effect adjusted for mediator*, *c’*	−1.018 (−2.587 to 0.551)	−2.458 (−6.333 to 1.417)	0.862 (−3.301 to 5.025)	−1.828 (−7.107 to 3.452)
	Mediated effect, *ab*	0.104 (−0.073 to 0.392)	0.671 (−0.383 to 1.91)	0.711 (−0.405 to 2.029)	0.789 (−0.449 to 2.306)

** The effect on the mediator (change of PSEQ from baseline to 4-month) from change of treatment, a, was 1.716 (−0.991 to 4.424) for all outcomes from baseline to 8- and 12-month.

Chronic tension type headache plus episodic migraine sub-group analysis
